# Exposure to ambient particulate matter and semen quality: the modifying role of socioeconomic status and lifestyle factors in chinese men

**DOI:** 10.1186/s12889-025-24600-4

**Published:** 2025-10-01

**Authors:** Weiling Liu, Lugang Zhao, Xizhong Yang, Lili Gu, Weiqi Liu, Huanjin Zhu

**Affiliations:** 1 Department of Clinical Laboratory, Foshan Fosun Chancheng Hospital, Foshan, Guangdong China; 2 Reproductive Medicine Research Centre, The Sixth Affiliated Hospital, Sun Yat-sen University, Guangzhou, Guangdong China; 3 Biomedical Innovation Center, The Sixth Affiliated Hospital, Sun Yat-sen University, Guangzhou, Guangdong China; 4 Department of Clinical Laboratory, The Maternal and Children Health Care Hospital (Huzhong Hospital) of Huadu, Guangzhou, Guangdong China

**Keywords:** Infertility, Particulate matter, Reproductive health, Semen quality, Socioeconomic status

## Abstract

**Background:**

The decline in birth rates and the increasing prevalence of infertility worldwide have become significant public health concerns. Research on the adverse effects of ambient air pollution, particularly particulate matter (PM), on male reproductive health has yielded somewhat inconsistent results.

**Methods:**

A cross-sectional study including 1,180 male participants aged 18–45 years was conducted from February 1, 2024, to January 31, 2025, at the Andrology Clinic of Foshan Fosun Chancheng Hospital, Foshan. To determine whether exposure to ambient PM_2.5_ (particles with aerodynamic diameter ≤ 2.5 μm), which constitutes a major component of ambient inhalable particulate matter (PM_10_, particles with aerodynamic diameter ≤ 10 μm) during different stages of sperm development is associated with alterations in semen quality, and to assess the potential modifying effects of socioeconomic status and lifestyle factors.

**Results:**

Exposure to ambient PM_2.5_ and PM_10_ during the 61–90 days before semen collection was associated with alterations in semen quality, with odds ratios (ORs) of 1.02 (95% CI, 1.00–1.04) and 1.01 (95% CI, 1.00–1.02) per 1 µg/m^3^ increase, respectively. Combined exposure to ambient PM and gaseous pollutants (e.g., CO and NO₂) further increased the risk of abnormal semen quality. Significant effect modification by education was observed for exposure to ambient PM_10_. Further analysis indicated that men with a lower socioeconomic status and unhealthy behaviours were more susceptible to the adverse effects of ambient PM exposure.

**Conclusion:**

Exposure to ambient particulate pollutants is associated with impaired semen quality. Men with a lower socioeconomic status and unhealthy lifestyles appear to be more vulnerable to these effects.

**Supplementary Information:**

The online version contains supplementary material available at 10.1186/s12889-025-24600-4.

## Background

The declining global birth rate and increasing problem of infertility have prompted the scientific community to conduct more in-depth research on male semen quality [1, 2]. Studies have shown that male factors account for approximately 50% of infertile couples [3]. A previous study reported that the average sperm concentration declined by 57% between 1980 and 2015 [4]. Levine et al. [5] further demonstrated that the decline in human sperm concentration has accelerated significantly since 2000, with the annual rate of decrease doubling compared with pre-millennium levels. A comprehensive analysis of 54 studies published between 1965 and 2015 revealed a 32.5% overall decline in the mean sperm concentration among European men [6]. An analysis of sperm donor eligibility in China between 2001 and 2015 revealed a marked decline in the proportion of qualified donors, dropping from 55.78% in 2001 to 17.80% in 2015 [7]. Therefore, the global decline in sperm quality represents a significant challenge for male reproductive health.

Epidemiological studies have shown a significant association between ambient air pollution exposure and semen quality [8, 9], with particulate matter (PM) [10–12]. Given that ambient fine particulate matter (PM_2.5_) constitutes the major fine fraction of ambient inhalable particulate matter (PM_10_) and is among the most widely monitored indicators of PM, this study focuses on ambient PM_2.5_ and PM_10_ as the primary exposure metrics. Importantly, PM_2.5_ is a component of PM_10;_ thus, these metrics are not independent. Herein, ambient PM_10_ refers to total inhalable particles, while ambient PM_2.5_ specifically denotes the fine fraction within PM_10_. These particles can infiltrate the human body through respiratory pathways, traversing the alveolar‒blood barrier to enter systemic circulation, potentially inducing widespread deleterious effects on the reproductive system [13]. A previous study reported a positive association between ambient PM_10_ exposure and forward sperm motility [14]. Additionally, a study of 200 adult men revealed that ambient PM_2.5_ exposure was related to decreased semen quality [15]. However, a study conducted among 228 men from three counties in the United States reported no statistically significant associations between sperm concentration, total sperm count, and ambient PM_2.5_ [16]. Therefore, further research across a wider range of geographical locations is needed to evaluate the specific impact of ambient PM on male reproductive health across different regions.

As a major industrial hub in the Pearl River Delta, Foshan has experienced gradual ambient air quality improvements but was ranked 18th among Guangdong cities in the January–November 2024 assessment. Its subtropical monsoon climate, with high heat and humidity, may lead to unique ambient PM dispersion patterns and health risks compared with drier or cooler regions. Investigating the link between ambient PM and semen quality here is crucial for informing region-specific public health strategies and addressing the data gap in southern China’s reproductive epidemiology.

## Methods

### Study design

We recruited 1,180 male participants aged 18–45 years from the andrology clinic of Foshan Fosun Chancheng Hospital between February 1, 2024, and January 31, 2025. All participants had resided in Foshan City for more than three months and provided at least one semen sample during the study period. Participants were excluded if they had conditions that could affect sperm quality, such as prior surgeries involving the testes or epididymis or vasectomies. Demographic and lifestyle data were collected via structured questionnaires (Table 1S). The study was approved by the hospital’s ethics committee (approval no. CYEC-LCYJ-2023074-PJ-20230927), and written informed consent was obtained from all participants.

### Semen quality assessment

After the participants’ abstinence duration was recorded, semen samples were collected via masturbation in sterile containers in a dedicated room. Following collection, the samples were liquefied in a 37 °C water bath for 30 min and analysed within 1 h. Following the WHO Laboratory Manual for the Examination and Processing of Human Semen (6th edition) [17], we evaluated semen parameters, including volume, pH, viability, concentration, total sperm count, and motility. Samples meeting all the following criteria were classified as normal controls: volume ≥ 1.5 mL, concentration ≥ 15 million/mL, total count ≥ 39 × 10^6^ per ejaculate, total motility ≥ 40%, progressive motility ≥ 32%, liquefaction time ≤ 60 min, pH ≥ 7.2, and viability ≥ 58%. Samples that failed any criterion were categorized as abnormal (defined as the disease group). All procedures were performed by trained technicians with regular laboratory quality control training to ensure data accuracy and consistency.

### Exposure assessment

In this study, we obtained 24-hour average concentrations of ambient PM_2.5_ and the corresponding PM_10_ (which contains PM_2.5_) from seven ground-level ambient air quality monitoring stations in Foshan City, covering the period from January 1, 2024, to December 31, 2024, through the National Urban Air Quality Real-Time Release Platform (The preprocessed dataset, which is updated daily, is publicly accessible at https://quotsoft.net/air). Based on the established timeline of human spermatogenesis from the WHO laboratory manual for the examination and processing of human semen (6th edition) [17], and in conjunction with previous epidemiological studies [18, 19], we estimated individual ambient PM_2.5_ and PM_10_ exposures using a time-weighted average method over the approximately 90-day sperm development window (encompassing approximately 64–74 days of spermatogenesis and approximately 7–14 days of epididymal maturation). Exposures were further assessed during three biologically defined subperiods aligned with key spermatogenic phases: the spermatogonial phase (days 61–90 before semen collection), corresponding to spermatogonial proliferation and mitosis; the meiotic phase (days 31–60 before semen collection), covering meiotic divisions of primary and secondary spermatocytes; and the sperm formation and maturation phase (days 0–30 before semen collection), encompassing spermiogenesis and epididymal maturation.

### Covariates

On the basis of previous studies [20, 21] and standardized questionnaires, we selected potential confounding factors, including age, education level, occupation, body mass index (BMI), income, smoking status, alcohol consumption, exercise, sleep duration, sugar-sweetened, cola consumption, coffee consumption, hyperuricaemia, and exposure to ambient gaseous pollutants such as sulphur dioxide (SO_2_), nitrogen dioxide (NO_2_), ozone (O_3_), and carbon monoxide (CO).

In this study, occupations was categorized into three groups—employee, self-employed, and other—based on participants self-reported primary occupational status. This classification did not evaluate specific occupational exposures (e.g., to chemicals, dust, metals, physical demands, or shift work). Education was classified into two categories: low education (high school or below) and high education (university or higher). BMI was defined according to a cut-off value of 25 kg/m²; participants with a BMI below 25 kg/m² were classified as normal, and those with a BMI of 25 kg/m² or higher were classified as overweight. Income was dichotomized using a threshold of 5000 RMB: ≤5000 RMB was classified as lower, and > 5000 RMB was classified as higher. Sleep duration was categorized using 7 h per day as the cut-off: ≤7 h was considered short, and > 7 h was considered long. Exercise, sugar-sweetened​ beverage consumption, cola consumption, and coffee consumption were all dichotomized based on weekly frequency and categorized as “yes” (at least once per week) or “no” (less than once per week). Smoking status was defined as a binary variable (yes/no). Participants classified as “yes” self-reported smoking any tobacco product—including but not limited to conventional cigarettes, e-cigarettes, and waterpipe—daily at any point within the 90 days preceding semen collection, consuming at least one cigarette (or equivalent product) per day during periods of active daily smoking. The classification criterion did not differentiate between (1) specific tobacco types, (2) smoking frequency patterns, (3) intensity measures (e.g., cigarettes per day, pack-years), or (4) former and never smokers (all non-daily smokers or those reporting no smoking within the preceding 90 days were categorized as “no”). Smoking status was based solely on self-report without biochemical verification (e.g., via urinary cotinine or exhaled carbon monoxide testing). Alcohol consumption, and hyperuricaemia were also assessed as binary variables (“yes” or “no”). The measurement and assessment methods for the concentrations of ambient SO_2_, NO_2_, O_3_, and CO were consistent with those used for ambient PM.

### Statistical analysis

Descriptive statistics were conducted on the participants’ baseline characteristics and levels of pollutant exposure. Categorical variables were summarized as frequencies, whereas continuous variables were reported as medians (P25, P75). Correlations among air pollutants were evaluated using Spearman’s rank correlation coefficients. We employed logistic regression models to assess the associations between exposure to ambient PM_2.5_ and PM_10_ across different the exposure windows (0–90 days, 0–30 days, 31–60 days, and 61–90 days) and semen quality. Ambient PM_2.5_ and PM_10_ exposures were modeled as continuous variables. We first assessed the independent effects of PM_2.5_ and PM_10_ in crude models. Subsequently, we adjustment for potential confounders, including age, occupation, BMI, sleep duration, and hyperuricaemia. To address potential confounding by gaseous pollutants, we further adjusted for coexposure to ambient SO_2_, NO_2_, O_3_, and CO in two-pollutant models. Additionally, to further explore the relationship between particulate pollution and semen quality, we employed restricted cubic splines with 4 knots (the 5th, 35th, 65th, and 95th percentiles) to flexibly model the associations of ambient PM with semen quality, adjusting for potential confounders in the models. Furthermore, conducted stratified analyses to evaluate potential differences in the effects of ambient PM on semen quality across various subgroups.

To assess the potential effects of socioeconomic status and health behaviours on the association between ambient PM exposure and semen quality, we conducted stratified analyses using random forest combined with unsupervised clustering. Seven key variables were selected for risk stratification: education, income, smoking, alcohol consumption, sugar-sweetened​, cola consumption, and coffee consumption (specifically defined as nonsmoking, nondrinking, no sugar-sweetened, no cola consumption, no coffee consumption, higher education, and higher income as low-risk indicators). We constructed a random forest model with 1,000 decision trees to generate a proximity matrix quantifying the multidimensional similarity between participants. The partitioning around medoids (PAM) algorithm was subsequently applied to cluster participants into three subgroups (k = 3) based on dissimilarity measures derived from the proximity matrix. A risk scoring system was developed by calculating the proportion of individuals with predefined high-risk features within each subgroup. The composite risk scores were used to stratify the subgroups into three tiers: high-risk (class 3), intermediate-risk (class 2), and low-risk (class 1).

All data analyses were performed using R version 4.2.0, primarily with the “rms”, and “randomForest” packages. ORs and 95% confidence intervals (CIs) were obtained by exponentiating the regression coefficients. It is essential to reiterate that all reported ORs for ambient PM_2.5_ and PM_10_ correspond to a 1 µg/m^3^ increase in concentration. Statistical significance was defined as a two-sided p value < 0.05.

## Results

Table 1 presents the baseline characteristics of the control and disease group participants. Age was comparable across both groups, with an interquartile range of 28–35 years for the control group and 28–36 years for the disease group; however, the disease group included a significantly higher proportion of patients aged > 35 years (27.1% vs. 20.3%, *p* < 0.001). No significant differences (*p* > 0.05) were detected in occupation; education; BMI; income; lifestyle factors (smoking, drinking, exercise, and sleep duration); beverage consumption (sugar-sweetened beverage consumption, cola consumption, and coffee consumption); hyperuricaemia; or ambient air pollutant levels (PM_2.5_, PM_10_, SO_2_, NO_2_, O_3_, and CO).

**Table 1:** Baseline demographics and clinical characteristics of the study participants in 2024–2025

Variable		Contrl group	Disease group	*p*-value
		(*n* = 770)	(*n* = 410)	
Age, *n* (%)	≤ 35 years	614 (79.7)	299 (72.9)	< 0.001
	> 35 years	156 (20.3)	111 (27.1)	
Education, *n* (%)	Low	359 (46.6)	183 (44.6)	0.554
	High	411 (53.4)	227 (55.4)	
Occupation, *n* (%)	Employee	71 (9.2)	40 (9.8)	0.509
	Freelancer	295 (38.3)	143 (34.9)	
	Other	404 (52.5)	227 (55.4)	
BMI, *n* (%)	Normal	488 (63.4)	263 (64.1)	0.843
	Overweight	282 (36.6)	147 (35.9)	
Income, *n* (%)	Lower	154 (20.0)	88 (21.5)	0.605
	Higher	616 (80.0)	322 (78.5)	
Sleep duration, *n* (%)	Short	582 (75.6)	310 (75.6)	0.999
	Long	188 (24.4)	100 (24.4)	
Smoking, *n* (%)	No	485 (63.0)	262 (63.9)	0.805
	Yes	285 (37.0)	148 (36.1)	
Alcohol consumption, *n* (%)	No	436 (56.6)	226 (55.1)	0.665
	Yes	334 (43.4)	184 (44.9)	
Exercise, *n* (%)	No	358 (46.5)	187 (45.6)	0.819
	Yes	412 (53.5)	223 (54.4)	
Sugar-sweetened, *n* (%)	No	198 (25.7)	96 (23.4)	0.424
	Yes	572 (74.3)	314 (76.6)	
Cola consumption, *n* (%)	No	440 (57.1)	230 (56.1)	0.777
	Yes	330 (42.9)	180 (43.9)	
Coffee consumption, *n* (%)	No	488 (63.4)	277 (67.6)	0.171
	Yes	282 (36.6)	133 (32.4)	
Hyperuricaemia, *n* (%)	No	703 (91.3)	365 (89.0)	0.244
	Yes	67 (8.7)	45 (11.0)	
Pollution, median (P25, P75)				
PM_2.5_, (µg/m^3^)		22.00 (16.32, 29.81)	22.06 (17.19, 29.78)	0.523
PM_10_, (µg/m^3^)		39.32 (27.68, 49.87)	39.37 (29.10, 49.87)	0.438
SO_2_, (µg/m^3^)		6.88 (6.23, 7.70)	6.95 (6.19, 7.61)	0.714
NO_2_, (µg/m^3^)		29.45 (24.48, 34.73)	29.64 (25.51, 34.52)	0.383
O_3_, (µg/m^3^)		58.70 (48.57, 61.32)	58.63 (48.00, 60.79)	0.073
CO, (mg/m^3^)		0.65 (0.58, 0.72)	0.65 (0.60, 0.72)	0.523

BMI body mass index, *P25* 25th percentiles, *P75* 75th percentiles, *PM2.5* particulate matter with aerodynamic diameter of ≤ 2.5 μm, *PM10* particulate matter with aerodynamic diameter of ≤ 10 μm of which PM2.5 is the fine fraction, *SO2* sulphur dioxide, *NO2* nitrogen dioxide, *O3* ozone, *CO* carbon monoxide

Table 2S shows significant positive correlations among most ambient air pollutants, specifically ambient PM_2.5_, PM_10,_ SO_2_, NO_2_, and CO—with the correlation between ambient PM_2.5_ and PM₁₀ reaching 0.996. This high correlation is expected because PM_2.5_ accounts for the major fraction of PM_10_. Conversely, ambient O_3_ displays weak negative correlations with other pollutants, especially CO, which has a correlation coefficient of −0.397. Notably, the correlation between ambient SO_2_ and O_3_ did not reach statistical significance (*p* > 0.05).

After adjusting for potential factors, in single-pollution exposure, our study revealed that increased exposure to ambient PM_2.5_ and PM_10_ during the 61–90 days was associated with changes in semen quality. Specifically, the OR of 1.02 (95% CI: 1.00–1.04) and 1.01 (95% CI: 1.00–1.02) per 1 µg/m^3^ increase, respectively. In dual-pollutant models, significant associations during days 61–90 were observed for ambient PM_2.5_ with CO (OR = 1.05, 95% CI: 1.01–1.08), ambient PM_10_ with NO_2_ (OR = 1.03, 95% CI: 1.00–1.07), and ambient PM_10_ with CO (OR = 1.02, 95% CI: 1.01–1.04) per 1 µg/m^3^ increase in each pollutant. Additionally, combined ambient PM_2.5_ and SO_2_ exposure throughout days 0–90 was associated with elevated risk (OR = 1.06, 95% CI: 1.01–1.12) per 1 µg/m^3^ increase (Table 2).

**Table 2:** Odds ratios (ORs) and 95% confidence intervals (CIs) for the associations of ambient PM_2.5_, PM_10_, and co-exposure with ambient gaseous pollutants on semen quality

Variable		Crude		Adjusted	
		OR (95%CI)	*p*-value	OR (95%CI)	*p*-value
Single-pollution					
PM_2.5_	lag 61–90 days	1.02 (1.01–1.04)	0.009	1.02 (1.00-1.04)	0.010
	lag 31–60 days	1.00 (0.99–1.02)	0.865	1.00 (0.99–1.02)	0.948
	lag 0–30 days	0.99 (0.98-1.00)	0.174	0.99 (0.98-1.00)	0.136
	lag 0–90 days	1.00 (0.99–1.02)	0.649	1.00 (0.99–1.02)	0.733
PM_10_	lag 61–90 days	1.01 (1.00-1.02)	0.006	1.01 (1.00-1.02)	0.008
	lag 31–60 days	1.00 (0.99–1.01)	0.746	1.00 (0.99–1.01)	0.672
	lag 0–30 days	1.00 (0.99-1.00)	0.338	1.00 (0.99-1.00)	0.277
	lag 0–90 days	1.00 (0.99–1.01)	0.633	1.00 (0.99–1.01)	0.719
Double-pollution					
PM_2.5_+SO_2_	lag 61–90 days	1.03 (0.99–1.07)	0.148	1.03 (0.99–1.07)	0.142
	lag 31–60 days	1.03 (0.99–1.06)	0.177	1.03 (0.99–1.06)	0.167
	lag 0–30 days	1.02 (1.00-1.05)	0.111	1.02 (0.99–1.05)	0.141
	lag 0–90 days	1.06 (1.01–1.12)	0.022	1.06 (1.01–1.12)	0.025
PM_2.5_+NO_2_	lag 61–90 days	1.05 (1.00-1.10)	0.071	1.04 (0.99–1.10)	0.080
	lag 31–60 days	1.02 (0.98–1.06)	0.347	1.02 (0.98–1.05)	0.388
	lag 0–30 days	0.97 (0.94–1.01)	0.108	0.97 (0.93–1.01)	0.100
	lag 0–90 days	1.01 (0.94–1.08)	0.786	1.01 (0.94–1.08)	0.814
PM_2.5_+O_3_	lag 61–90 days	1.02 (1.01–1.04)	0.009	1.02 (1.00-1.04)	0.010
	lag 31–60 days	1.00 (0.99–1.02)	0.860	1.00 (0.99–1.02)	0.947
	lag 0–30 days	0.99 (0.98–1.01)	0.227	0.99 (0.98-1.00)	0.178
	lag 0–90 days	1.00 (0.98–1.02)	0.903	1.00 (0.98–1.02)	0.975
PM_2.5_+CO	lag 61–90 days	1.04 (1.01–1.08)	0.015	1.05 (1.01–1.08)	0.014
	lag 31–60 days	0.97 (0.93-1.00)	0.080	0.97 (0.93-1.00)	0.070
	lag 0–30 days	0.95 (0.92–0.98)	0.002	0.95 (0.92–0.98)	0.003
	lag 0–90 days	0.91 (0.83–0.99)	0.027	0.91 (0.83–0.99)	0.029
PM_10_ + SO_2_	lag 61–90 days	1.02 (1.00-1.04)	0.098	1.02 (1.00-1.04)	0.101
	lag 31–60 days	1.00 (0.98–1.02)	0.926	1.00 (0.98–1.02)	0.890
	lag 0–30 days	1.01 (1.00-1.03)	0.042	1.01 (1.00-1.03)	0.054
	lag 0–90 days	1.03 (1.00-1.06)	0.033	1.03 (1.00-1.06)	0.037
PM_10_ + NO_2_	lag 61–90 days	1.03 (1.00-1.07)	0.026	1.03 (1.00-1.07)	0.032
	lag 31–60 days	1.00 (0.97–1.02)	0.701	0.99 (0.97–1.02)	0.654
	lag 0–30 days	0.99 (0.97–1.02)	0.584	0.99 (0.97–1.02)	0.554
	lag 0–90 days	1.01 (0.96–1.06)	0.671	1.01 (0.96–1.06)	0.716
PM_10_ + O_3_	lag 61–90 days	1.01 (1.00-1.02)	0.006	1.01 (1.00-1.02)	0.008
	lag 31–60 days	1.00 (0.99–1.01)	0.735	1.00 (0.99–1.01)	0.657
	lag 0–30 days	1.00 (0.99-1.00)	0.412	1.00 (0.99-1.00)	0.339
	lag 0–90 days	1.00 (0.99–1.01)	0.862	1.00 (0.99–1.01)	0.937
PM_10_ + CO	lag 61–90 days	1.03 (1.01–1.04)	0.010	1.02 (1.01–1.04)	0.010
	lag 31–60 days	0.98 (0.96–0.99)	0.010	0.98 (0.96–0.99)	0.009
	lag 0–30 days	0.98 (0.97-1.00)	0.071	0.98 (0.97-1.00)	0.075
	lag 0–90 days	0.96 (0.92-1.00)	0.083	0.96 (0.92–1.01)	0.083

*OR* Odds ratios, *95%CI* 95% confidence intervals, *PM2.5* particulate matter with aerodynamic diameter of ≤ 2.5 μm, *PM10* particulate matter with aerodynamic diameter of ≤ 10 μm, of which PM2.5 is the fine fraction, *SO2* sulphur dioxide, *NO2* nitrogen dioxide, *O3* ozone, *CO* carbon monoxide

a crude model without adjustment for covariates

b adjusted for covariates, including age, occupation, BMI, sleep duration, and hyperuricaemia

**Fig. 1:**
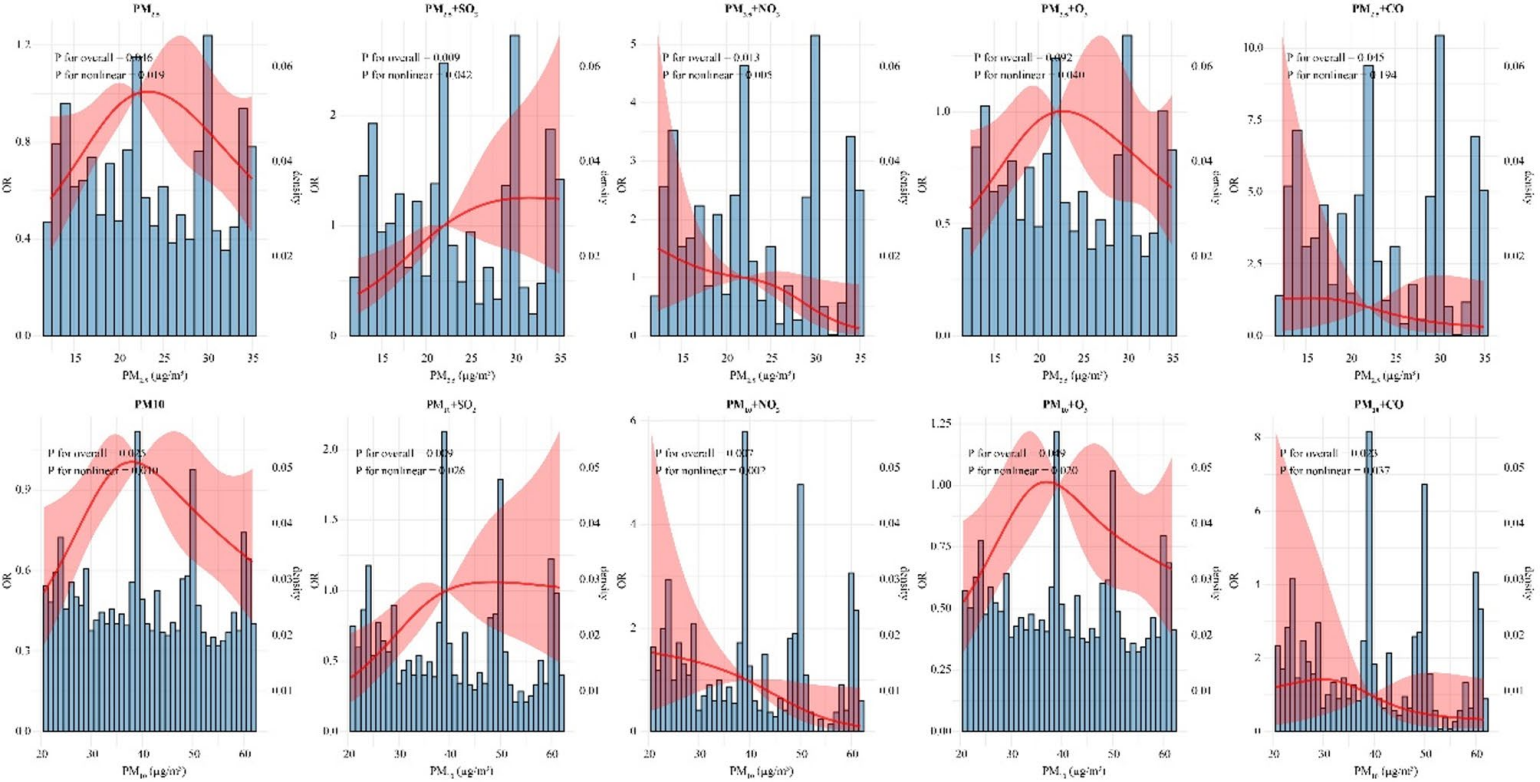
Restricted cubic spline curves for ambient PM_2.5_ and PM_10_ exposure and semen quality. The solid line represents the OR, while the dashed area indicates the 95% CI. The baseline is set at the minimum level for PM_2.5_ and PM_10_, with data points set at the 5th, 35th, 65th and 95th percentiles for both PM_2.5_ and PM_10_. *OR*, Odds ratios. *95%CI*, 95% confidence intervals. *PM*_*2.5*_, particulate matter with aerodynamic diameter of ≤ 2.5 μm. *PM*_*10*_, particulate matter with aerodynamic diameter of ≤ 10 μm, of which PM_2.5_ is the fine fraction. *SO*_*2*_, sulphur dioxide. *NO*_*2*_, nitrogen dioxide. *O*_*3*_, ozone. *CO*, carbon monoxide

As shown in Fig. 1, a nonlinear association was observed between exposure to ambient PM_2.5_ and PM_10_ (includes PM_2.5_) and semen quality as well as their combinations with ambient gaseous pollutants (PM_2.5_+SO_2_, PM_2.5_+NO_2_, PM_2.5_+O_3_, PM_10_ + SO_2_, PM_10_ + NO_2_, PM_10_ + O_3_, and PM_10_ + CO) during the 0–90 days.

**Fig. 2:**
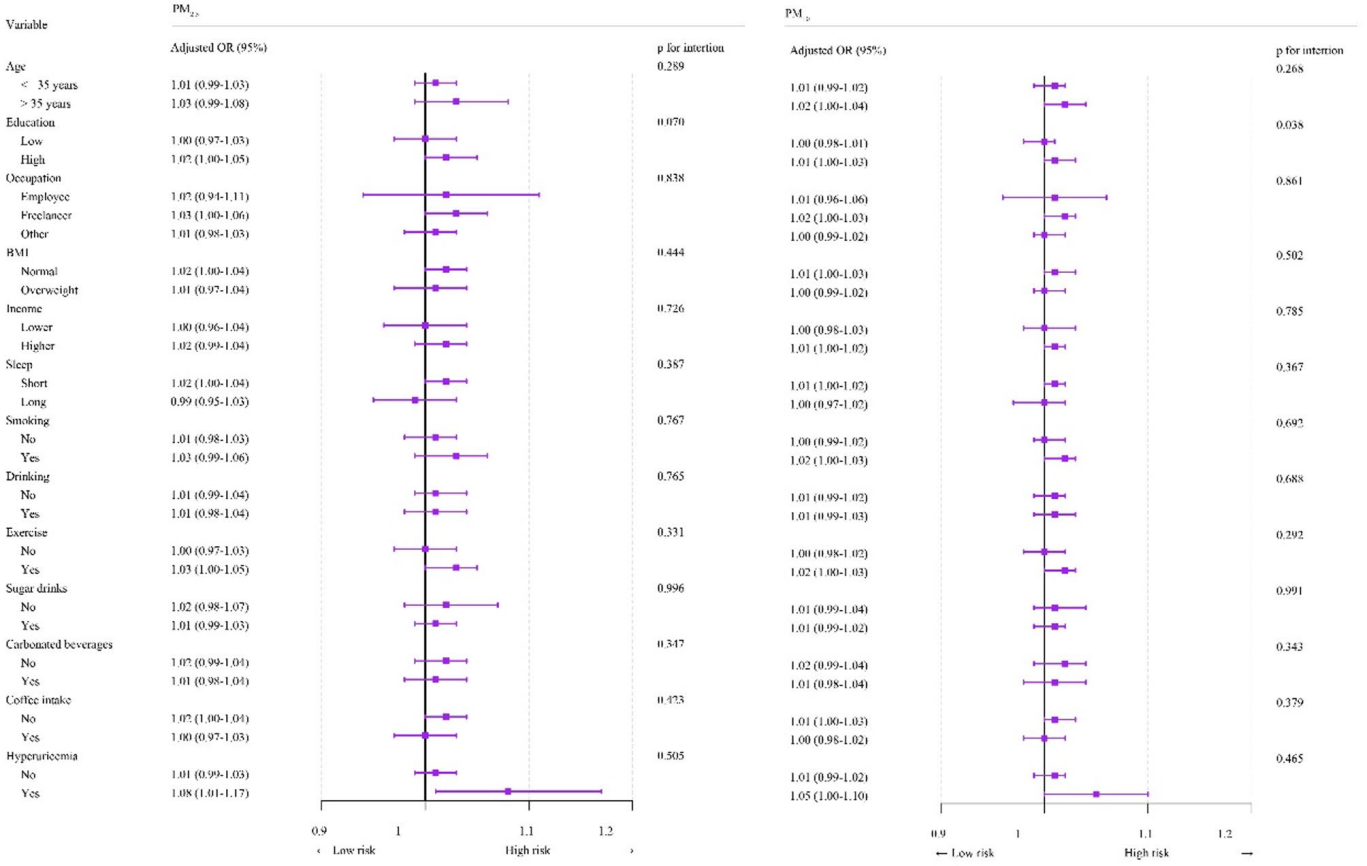
Subgroup analyses of the association between ambient PM_2.5_ and PM_10_ (includes PM_2.5_) exposure and semen quality. Adjusted for covariates, including age, occupation, BMI, sleep duration, and hyperuricaemia. *OR*, Odds ratios. *95%CI*, 95% confidence intervals. *PM*_*2.5*_, particulate matter with aerodynamic diameter of ≤ 2.5 μm. *PM*_*10*_, particulate matter with aerodynamic diameter of ≤ 10 μm, of which PM_2.5_ is the fine fraction

As shown in Fig. 2, ambient PM_2.5_ exposure showed no statistically significant interaction with age, education level, occupation, BMI, income, sleep duration, smoking, alcohol consumption, exercise, sleep duration, sugar-sweetened, cola consumption, coffee consumption, or hyperuricaemia (P for interaction > 0.05). In contrast, ambient PM_10_ exposure showed a significant interaction with education level (P for interaction < 0.05).

**Fig. 3:**
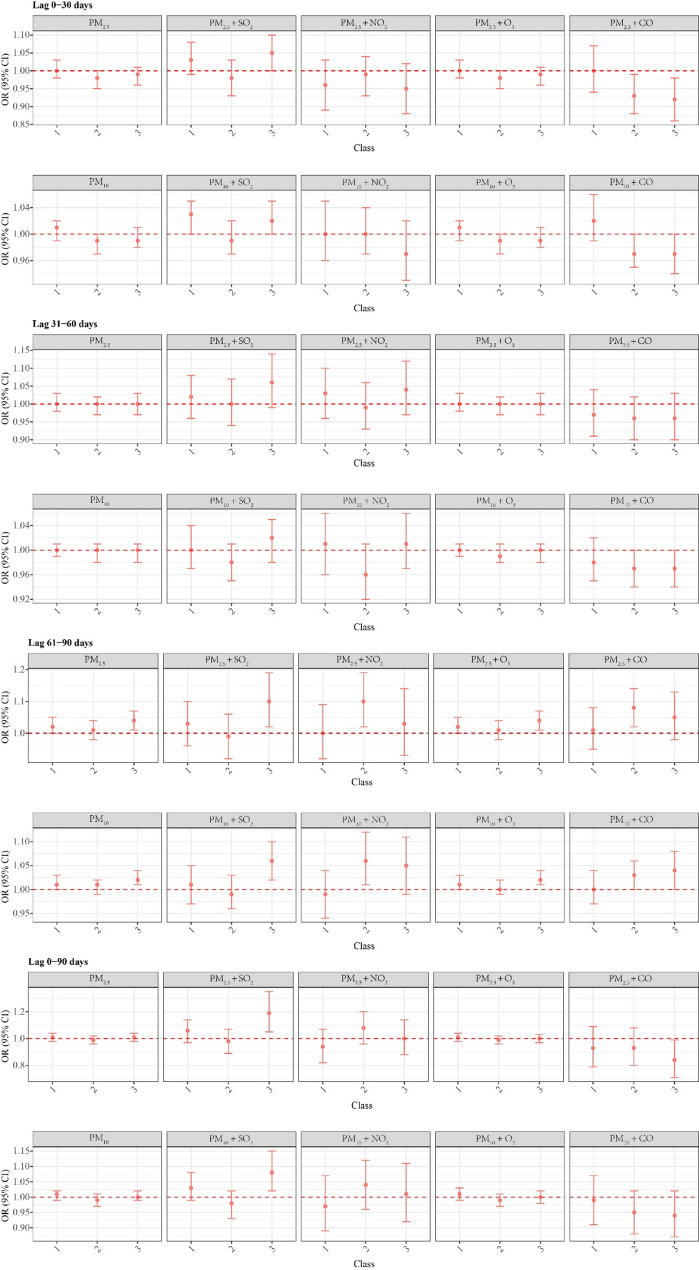
Stratified effects of ambient PM exposures on semen quality risk by socioeconomic status and behavioral risk groups. Adjusted for covariates, including age, occupation, BMI, sleep duration, and hyperuricaemia. *OR*, Odds ratios. *95%CI*, 95% confidence intervals. *PM*_*2.5*_, particulate matter with aerodynamic diameter of ≤ 2.5 μm. *PM*_*10*_, particulate matter with aerodynamic diameter of ≤ 10 μm, of which PM_2.5_ is the fine fraction. *SO*_*2*_, sulphur dioxide. *NO*_*2*_, nitrogen dioxide. *O*_*3*_, ozone. *CO*, carbon monoxide

Our analysis revealed differential effects of ambient PM on semen quality across socioeconomic status and health behaviours. During the 0–30 days, combined ambient PM_2.5_+SO_2_ exposure significantly increased the risk of poor semen quality in the high-risk group, while ambient PM_10_ + SO_2_ exposure increased the risk in both the low- and high-risk groups. In the 61–90 day period, ambient PM_2.5_ and PM_10_ alone increased the risk in both the low- and high-risk groups, whereas combined exposures showed stratified associations: ambient PM_2.5_+SO_2_ and PM_10_ + SO_2_ impacted the high-risk group; ambient PM_2.5_+NO_2_ and PM_10_ + NO_2_ affected the moderate-risk group; ambient PM_2.5_+O_3_ and PM_10_ + O_3_ increased the risk in the low- and high-risk groups; and CO combinations (PM_2.5_+CO for moderate-risk; PM_10_ + CO for moderate/high-risk) exhibited distinct patterns. Over the 0–90 days, ambient PM_2.5_+SO_2_ and PM_10_ + SO_2_ remained specifically associated with high-risk group susceptibility (Fig. 3).

## Discussion

This study aimed to investigate the potential association between exposure to ambient PM and semen quality among men in Foshan. We found that exposure to ambient PM during the 61–90 days spermatogenic window was positively associated with changes in semen quality. Furthermore, coexposure to ambient PM and gaseous pollutants (NO_2_, O_3_, and CO) during the same developmental period significantly increased the risk of abnormal semen quality. Stratified analyses by socioeconomic status and health behaviours indicated that men of lower socioeconomic status and those with poorer lifestyle habits were more susceptible to semen quality impairment following ambient PM exposure during the 61–90 days window. Furthermore, to the best of our knowledge, this is the first study conducted in China to investigate the roles of socioeconomic status and lifestyle habits in the association between ambient PM exposure and semen quality.

The biological mechanisms by which ambient PM affects semen quality remains to be fully elucidated. Current evidence suggests that exposure to ambient PM induces excessive generation of reactive oxygen species, such as peroxides, hydrogen peroxide, and hydroxyl radicals, within body tissues [22–24]. In vitro studies have demonstrated that atmospherically aged ambient PM induces greater oxidative stress and air pollution exposure levels than its freshly emitted counterparts do [25]. Studies by Liu et al. [26] have demonstrated that ambient PM can induce oxidative stress, leading to apoptosis of testicular Sertoli cells and disruption of the blood‒testis barrier, ultimately resulting in impaired spermatogenesis in males. Furthermore, Zheng et al. [27] demonstrated through both in vivo and in vitro studies that ambient PM_2.5_ exposure may induce male reproductive dysfunction by triggering ferroptosis and modulating the HIF-1α signalling pathway. Our findings demonstrate that exposure to ambient PM during the 61–90 days spermatogenic window is significantly associated with altered semen quality. These results are consistent with those of previous studies, collectively highlighting the adverse effects of ambient PM exposure on early-stage semen quality parameters [28–30]. Notably, combined exposure to ambient PM and SO₂ throughout the 0–90 days spermatogenic window significantly elevated the risk of impaired semen quality. These findings suggest that coexposure to ambient gaseous pollutants may potentiate the reproductive health risks of ambient PM, potentially through exacerbated oxidative damage and/or epigenetic modifications [26, 31]. The observed nonlinear exposure‒response relationship between ambient PM exposure and semen quality further underscores the complexity of air pollution interactions, potentially reflecting threshold effects or dynamic antagonistic/synergistic interactions between pollutants [32]. These findings provide mechanistic insights into the association between ambient PM and semen quality, wherein particulate-bound components (e.g., heavy metals and polycyclic aromatic hydrocarbons) can impair Sertoli cell integrity and sperm mitochondrial function, whereas concomitant ambient gaseous pollutant exposure may exacerbate systemic inflammatory responses [18, 33]. Therefore, these findings underscore the need for regulatory policies specifically targeting ambient PM and its synergistic interactions with gaseous pollutants to mitigate potential risks to male reproductive health.

In studies investigating the association between ambient PM exposure and semen quality, the role of subgroup factors has consistently been a key research focus [34, 35]. Studies have demonstrated that smoking is closely associated with semen quality [36, 37], Moreover, lifestyle factors such as alcohol consumption and physical activity also influence semen quality [38–40]. Furthermore, the association between ambient PM exposure and semen quality may also be influenced by individual characteristics, including age, BMI, educational attainment, and income level [28, 41, 42]. Contrary to these expectations, our analysis revealed no statistically significant interactions between ambient PM_2.5_ exposure and semen quality for age, education level, occupation, BMI, income, sleep duration, smoking, alcohol consumption, physical activity, sugar-sweetened intake, cola intake, coffee consumption, or hyperuricaemia. Notably, ambient PM_10_ exposure showed a significant interaction with education level, though no other subgroups demonstrated effect modification. These discrepancies may be attributed to differences in study design, sample characteristics, exposure assessment methods, and statistical approaches. Future research should further investigate the role of these subgroup factors under varying environmental exposure conditions to better understand the potential impact of ambient PM on semen quality.

Epidemiological studies indicate that individuals with a lower socioeconomic status tend to reside near major roadways and industrial areas, where pollution levels are typically higher, leading to prolonged exposure to elevated concentrations of ambient PM, which is significantly associated with an increased risk of abnormal semen quality [43–45]. Considering that people spend most of their time indoors, the impact of indoor air pollution on sperm health is particularly critical. Indoor air quality, however, is significantly influenced by outdoor conditions. Studies show that a substantial portion of indoor pollutants originates from outdoors, with up to 40% of outdoor particulate matter penetrating indoors even with windows closed [46]. Individuals of lower socioeconomic status often live in homes with poor sealing and inadequate ventilation systems, which greatly reduce the ability to block and remove outdoor pollutants, allowing them to accumulate more easily. As a result, this group is not only more likely to reside in areas with high outdoor pollution but also lacks effective indoor protective measures, facing a “double burden of exposure.” This combined effect is especially pronounced in developing countries with weaker air quality regulations, placing them at higher risk of impaired sperm quality. Furthermore, disadvantaged populations often face greater challenges in adopting healthy lifestyle practices, including for example, higher rates of smoking and alcohol consumption. The cumulative effect of these factors may further compromise semen quality [47, 48]. In contrast, individuals with a higher socioeconomic status are more likely to adopt healthier lifestyles and have better access to quality health care services, which may help mitigate the negative health effects of ambient pollution [49, 50]. Our study examining the exposure‒response relationship between ambient PM exposure and semen quality across different socioeconomic and lifestyle groups revealed significantly increased risks of semen quality abnormalities in the medium- and high-risk groups. Notably, during the 61–90 days, the high-risk group exposed to both ambient particulate and gaseous pollutants presented markedly elevated risks. These findings suggest that individuals with a lower socioeconomic status and poorer lifestyle habits may be more vulnerable to the toxic effects of ambient PM, whereas healthier lifestyles and better access to medical resources may partially mitigate this susceptibility.

This study has several limitations. First, the population was recruited only from Foshan City and may not be representative of other regions or the general population due to demographic and environmental specificities. Second, the exposure assessment was limited: it relies on averages from fixed monitoring stations and does not account for individual time-activity patterns (e.g., indoor/outdoor activities, occupation, commuting), nor does it measure personal actual indoor PM concentrations, potentially leading to exposure misclassification and biased effect estimates. furthermore, ultrafine particles and coarse particles (PM_2.5−10_, i.e., the fraction of PM_10_ not included in PM_2.5_) were not addressed due to data constraints. Thirdly, information on key confounders was not collected in detail. For example, smoking status was assessed only using a crude binary classification, and relied solely on self-reporting without biomarker validation. Occupational categories were oversimplified into only three groups, failing to capture specific exposures to reproductive toxicants such as dusts, gases, metals, night shifts, or sedentary work [51]. Critically, we did not collect any data on exposure to bisphenol A, a potent endocrine disruptor with strong evidence linking it to impaired semen quality [52, 53]. This lack of heterogeneity may be attributed to limitations in the study design, insufficient sample size, imprecise exposure assessment, measurement error in key variables, and the statistical methods used. Future studies should incorporate longitudinal follow-up, more precise individual exposure assessment, detailed smoking history (including type, intensity, and cessation status), and quantitative assessment of occupational exposures to better clarify the effects of ambient PM on semen quality in different populations.

## Conclusions

Our findings indicate that exposure to ambient PM adversely affects semen quality in men, particularly during the 61–90 days before semen collection. Furthermore, men with a lower socioeconomic status and poorer lifestyle habits are more susceptible to the detrimental effects of particulate pollution on semen quality, highlighting the importance of minimizing exposure to such pollutants in this vulnerable population.

## Supplementary Information

Supplementary Material 1.

## Data Availability

The data that support the findings of this study are available from the corresponding author (Weiling Liu), upon reasonable request.
