# Supplementary material for: Exposure to ambient particulate matter and semen quality: the modifying role of socioeconomic status and lifestyle factors in chinese men
Source: BMC Public Health. 2025 Oct 1;25:3263. doi: 10.1186/s12889-025-24600-4 (PMC12487108; doi:10.1186/s12889-025-24600-4)
Supplement: Supplementary file 1 — Supplementary Material 1. [file 12889_2025_24600_MOESM1_ESM.docx]

Supplemental Material


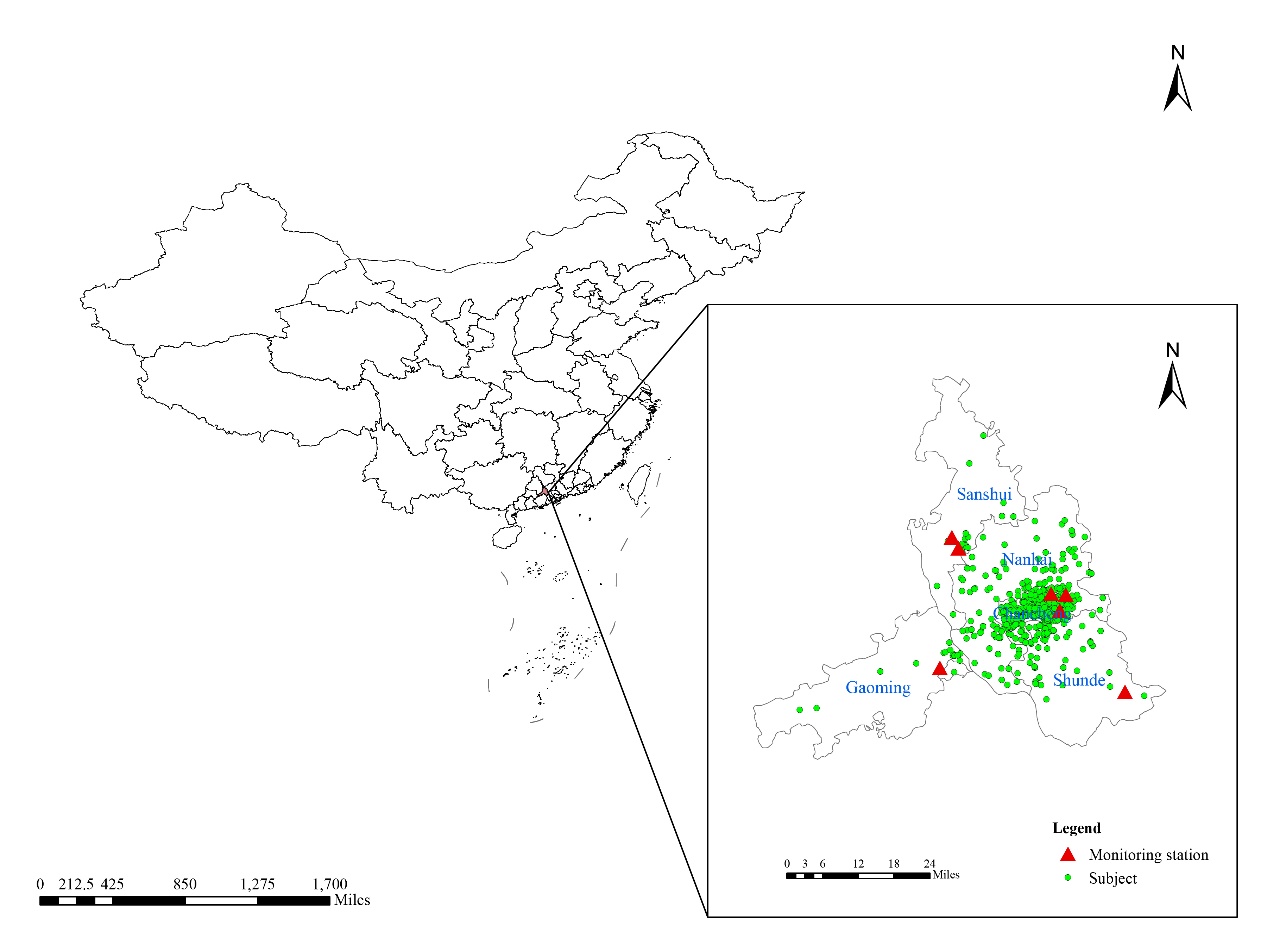


Figure 1S Geographical Distribution of Study Subjects and Monitoring Stations in Foshan

Table 1S Survey Questionnaire

| Dear Participant,  Greetings! This survey is conducted to better understand the factors influencing semen quality. All information provided will be kept strictly confidential. By participating in this survey, you are indicating your informed consent. Please answer the questions based on your actual situation. Thank you for your participation! |
| --- |
| Age: _________ years |
| Height: _________ cm. Weight: _________ kg |
| Ethnicity: □ Han □ Other (please specify): _________ |
| Education level: □ High school or below. □ College   - Bachelor's degree or higher |
| Days of abstinence: □ 3–4 days □ 5–7 days □ 7 days |
| Occupation: □ Employee □ Freelancer  □ Other (please specify): _________ |
| Smoking status: □No □ 1–10 cigarettes per day  □ 10 cigarettes per day |
| Alcohol consumption: □ No □ Occasional drinking □ Frequent drinking |
| Income: □ ≤5,000 RMB/month □ 5,000–10,000 RMB/month  □ ≥10,000 RMB/month |
| Exercise frequency: □ None □ 3–5 times per week □ >5 times per week |
| Sleep time: □ 8 hours per day □ 6–7 hours per day □ <6 hours per day |
| Distance from highway or ring road: □ <1 km □ 1–5 km □ >5 km |
| Sugar-sweetened beverage intake: □ None □1–2 times per week  □ ≥3 times per week |
| Cola consumption: □None □1–2 times per week □≥3 times per week |
| Coffee consumption: □None □1–2 times per week  □ ≥3 times per week |
| History of diabetes: □ No □ Yes |
| History of hypertension: □ No □ Yes |
| History of hyperuricemia: □ No □ Yes |
| Related Surgeries or Conditions:  □ None  □ Urethral surgery  □ Testicular surgery  □ Epididymal surgery  □ Vasectomy  □ Azoospermia |
| Current Residential Address: |

Table 2S Spearman correlation analysis of ambient PM_2.5_, PM_10_, SO_2_, NO_2_, O_3_, and CO during the study period

|  | PM_2.5_ | PM_10_ | SO_2_ | NO_2_ | O_3_ | CO |
| --- | --- | --- | --- | --- | --- | --- |
| PM_2.5_ | 1 |  |  |  |  |  |
| PM_10_ | 0.996^*^ | 1 |  |  |  |  |
| SO_2_ | 0.934^*^ | 0.920^*^ | 1 |  |  |  |
| NO_2_ | 0.970^*^ | 0.980^*^ | 0.880^*^ | 1 |  |  |
| O_3_ | -0.248^*^ | -0.228^*^ | -0.052 | -0.103^*^ | 1 |  |
| CO | 0.982^*^ | 0.974^*^ | 0.892^*^ | 0.924^*^ | -0.397^*^ | 1 |

^*^: *p* < 0.05

*PM_2.5_*, particulate matter with aerodynamic diameter of ≤2.5μm. *PM_10_*, particulate matter with aerodynamic diameter of ≤ 10μm, , of which PM_2.5_ is the fine fraction. *SO_2_*, sulphur dioxide. *NO_2_,* nitrogen dioxide. *O_3_*, ozone. *CO*, carbon monoxide.
